# In Vivo Biocompatibility Investigation of an Injectable Calcium Carbonate (Vaterite) as a Bone Substitute including Compositional Analysis via SEM-EDX Technology

**DOI:** 10.3390/ijms23031196

**Published:** 2022-01-21

**Authors:** Ronald E. Unger, Sanja Stojanovic, Laura Besch, Said Alkildani, Romina Schröder, Ole Jung, Caroline Bogram, Oliver Görke, Stevo Najman, Wolfgang Tremel, Mike Barbeck

**Affiliations:** 1 Repair-Lab, Institute of Pathology, University Medical Center of the Johannes Gutenberg University, 55131 Mainz, Germany; runger@uni-mainz.de; 2 Department of Biology and Human Genetics, Faculty of Medicine, University of Niš, 18108 Niš, Serbia; sanja.genetika.nis@gmail.com (S.S.); stevo.najman@gmail.com (S.N.); 3 Scientific Research Center for Biomedicine, Department for Cell and Tissue Engineering, Faculty of Medicine, University of Niš, 18108 Niš, Serbia; 4 Institute of Inorganic Chemistry and Analytical Chemistry, Johannes Gutenberg-University of Mainz, 55128 Mainz, Germany; labesch@uni-mainz.de (L.B.); r.schroeder@uni-mainz.de (R.S.); tremel@uni-mainz.de (W.T.); 5 BerlinAnalytix GmbH, Ullsteinstrasse 108, 12109 Berlin, Germany; Said.alkildani@berlinanalytix.com (S.A.); caroline.bogram@berlinanalytix.com (C.B.); 6 Clinic and Policlinic for Dermatology and Venereology, University Medical Center Rostock, 18057 Rostock, Germany; ole.tiberius.jung@googlemail.com; 7 Institute of Materials Science and Technology, Chair of Advanced Ceramic Materials, Technical University Berlin, Hardenbergstr. 40, 10623 Berlin, Germany; o.goerke@tu-berlin.de

**Keywords:** injectable bone substitutes, bone tissue engineering, guided bone regeneration, EDX mapping, calcium carbonate, vaterite, biomaterial-induced multinucleated giant cells

## Abstract

Injectable bone substitutes (IBS) are increasingly being used in the fields of orthopedics and maxillofacial/oral surgery. The rheological properties of IBS allow for proper and less invasive filling of bony defects. Vaterite is the most unstable crystalline polymorph of calcium carbonate and is known to be able to transform into hydroxyapatite upon contact with an organic fluid (e.g., interstitial body fluid). Two different concentrations of hydrogels based on poly(ethylene glycol)-acetal-dimethacrylat (PEG-a-DMA), i.e., 8% (*w*/*v*) (VH-A) or 10% (*w*/*v*) (VH-B), were combined with vaterite nanoparticles and implanted in subcutaneous pockets of BALB/c mice for 15 and 30 days. Explants were prepared for histochemical staining and immunohistochemical detection methods to determine macrophage polarization, and energy-dispersive X-ray analysis (EDX) to analyze elemental composition was used for the analysis. The histopathological analysis revealed a comparable moderate tissue reaction to the hydrogels mainly involving macrophages. Moreover, the hydrogels underwent a slow cellular infiltration, revealing a different degradation behavior compared to other IBS. The immunohistochemical detection showed that M1 macrophages were mainly found at the material surfaces being involved in the cell-mediated degradation and tissue integration, while M2 macrophages were predominantly found within the reactive connective tissue. Furthermore, the histomorphometrical analysis revealed balanced numbers of pro- and anti-inflammatory macrophages, demonstrating that both hydrogels are favorable materials for bone tissue regeneration. Finally, the EDX analysis showed a stepwise transformation of the vaterite particle into hydroxyapatite. Overall, the results of the present study demonstrate that hydrogels including nano-vaterite particles are biocompatible and suitable for bone tissue regeneration applications.

## 1. Introduction

Most tissues in the human body have the capacity to repair themselves via physiological processes. However, at times, these capacities can be limited [1]. In bone tissue, the capacity to repair larger defects (so-called critical-size defects), for example caused by extensive traumatic damage or disease, can be too substantial to allow for a normal physiological repair to take place [2,3]. This type of defect is often a challenging problem encountered in maxillofacial and oral surgery for Guided Bone Regeneration (GBR). GBR is a widely used technique in surgical dentistry prior to prosthetic implantation. The method aims to enhance natural bone regrowth at the defect site via the implantation of so-called bone substitute materials (BSM), which act as a scaffold structure for host cells to grow on [4,5,6]. This process, known as bone tissue regeneration, has clinically been used for several decades.

As a gold standard, bone tissue from the same patient is harvested and implanted in the defect site. However, this so-called autograft is often associated with additional complications due to the need for a second invasive surgery to obtain healthy bone, often resulting in donor site morbidity and low or compromised availability in geriatric individuals [2]. Alternatives to autografts can be sourced from other humans in the form of so-called allografts that are mostly obtained from donated tissue from hip arthroplasty surgeries [7]. Another frequently used bone substitute material group can be obtained from a number of animals, i.e., so-called xenografts mostly from bovine and porcine sources [8]. In the last 20 years, a strong focus has been on the creation of bone-like materials via synthetic means, and these are known as alloplastic grafts [4].

The alloplastic materials have the enormous advantage of being safe as the manufacturing process of this group of materials do not require a purification or decellularization process [9]. However, the goal is to continually optimize synthetic BSM to achieve the best and most natural bone regeneration process. To achieve this goal, a BSM must meet certain requirements for its successful application in GBR or similar techniques [2,9]. The biomaterial must be biocompatible, it should not elicit a persistent exaggerated pro-inflammatory immune response, it must provide a biomimicking micro-milieu to bone tissue and should be biodegradable at a rate parallel to natural bone regrowth [9]. To create such as a micro-milieu for bone tissue regeneration a BSM must provide an optimal combination of physicochemical properties to induce a suitable level of bioactivity after implantation that encourages the growth of new bone [2,9]. While natural BSM (such as the above-mentioned natural autograft, allograft or xenograft materials) only allow for minimal adaption of their properties due to the different purification processes used, the physicochemical characteristics of alloplastic BSM can easily be modified. This characteristic makes the use and manufacture of such materials highly favorable compared to using the various natural materials. Therefore, this material class is of special interest due to its predictable manufacturing process resulting in well controllable physicochemical material characteristics such as the chemical composition or the pore size and distribution [2,9]. The entirety of these BSM properties have been shown to have a decisive influence on the material-mediated bone healing process based on the cellular reactions [10,11,12].

However, studies in this area are still in the early stages. It is not clear what difference or small changes in physicochemical material these properties have on the healing process for bone tissue regeneration. Much information is still lacking on how the physicochemical material properties influence molecular cascades such as cell growth, healing steps, and bone tissue regeneration, especially when used in different application sites. Moreover, it has been shown that a particular biomaterial can induce a specific unique immune response, which, in the optimal case, can further support the molecular processes of tissue healing [13,14]. It has been shown that macrophages and their pro- and anti-inflammatory subtypes are key players in this immunological cascade known as “foreign body reaction to biomaterials” [15]. In addition, it has been found that in their fused end stage, the biomaterial-associated multinucleated giant cells (BMGC) that are also found within the implantation beds of different biomaterials, and especially those adhered to BSM, have comparable properties similar to their mononuclear precursors [13,16]. Both cell types can express a broad variety of pro- and anti-inflammatory signaling molecules dependent on the sum of physicochemical biomaterial properties that can support (bone) tissue healing cascades but can also lead to implant failures such as fibrotic encapsulation [17]. Thus, it is of importance not only to examine the perfunctory tissue compatibility of a biomaterial but also the immunologic response of the host tissue in order to develop optimal materials for use as BSM. Therefore, properties of materials must be determined which can influence supportive processes such as the implantation bed vascularization via induction of the expression of the vascular endothelial growth factor (VEGF) [18]. Altogether, it is assumed that the anti-inflammatory macrophage subtype and its respective expression profile mainly contributes to (bone) tissue regeneration [19]. Thus, materials that trigger an overall anti-inflammatory tissue response appear to be favorable.

The most common and commercially available synthetic BSM are based on calcium phosphates, which include hydroxyapatite (HA), beta-tricalcium phosphates (β-TCP) and biphasic calcium phosphates (i.e., a combination of HA and β-TCP) [20]. Interestingly, HA crystals make up most of the inorganic part of human bone tissue and it has been shown that during initial bone mineralization, amorphous calcium phosphates are the precursor compound of crystalline HA (cHA) found in mature and fully developed bone [9,21]. However, one of the most challenging aspects of using calcium phosphates clinically is their slow resorption rate that does not run in tandem with bone regrowth, hindering the regeneration process [9].

Vaterite is the metastable phase (crystalline polymorph) of calcium carbonate [21]. Vaterite is also the least stable crystalline anhydrous polymorph of calcium carbonate, which has the reactivity to transform into HA [21,22]. Moreover, phosphate is attracted by the positive surface charge and appears to interact with the calcium [23]. Briefly, vaterite can be synthesized by combining calcium chloride tetrahydrate and sodium bicarbonate in ethylene glycol [21]. When synthetic vaterite was incubated in simulated body fluid (SBF) for 24 h, 48 h and 72 h to investigate its transformation into cHA at the 72 h timepoint, over 35% of the vaterite was transformed into cHA [21]. In addition, when the immunological reaction of endothelial cells to the vaterite was examined via the CAM-EIA assay in vitro, no induction of these factors was observed. Furthermore, endothelial cells will respond to endotoxin via the expression of E-selectins, which are adhesion molecules that retard immunological cells (macrophages, neutrophils, etc.) in the bloodstream as they pass a site of infection by a pathogen or foreign object in the tissue next to the blood vessel [24]. Endothelial cells seeded on vaterite did not express E-selectins on their surface membranes. Thus, vaterite is highly biocompatible for endothelial cells.

Another challenging aspect of using calcium phosphates clinically is their brittleness and this compromises their manageability when applied by physicians [9,25,26]. Thus, BSM are combined with natural biodegrading hydrogels to create injectable bone substitutes (IBS) that are easy to handle clinically, as well as to create an optimal filling capability in the case of fracture or defect cavities and interspaces between the implants and the implant beds [26,27]. Hydrogels in IBS are also biodegradable, which allows for bone regrowth in a creeping substitution fashion, where the resorption rate of the biomaterial matches the growth of new bone [27]. This ultimately allows a restitutio ad integrum of the defective site. In vivo, IBS show adequate integration and a resorption rate that runs in parallel to GBR [27]. A clinical trial applying IBS for GBR purposes showed complete regrowth of bone tissue resulting in a successful prosthetic implantation [26]. Injectables can be highly tailored by controlling the viscosity of the hydrogel, the hydrogel to bone substitute ratio and the cross-linkage [28,29].

The present paper investigates different ratios of poly(ethylene glycol)-acetal-dimethacrylat (PEG-a-DMA) hydrogels, i.e., 8% (*w*/*v*) (VH-A) or 10% (*w*/*v*) (VH-B), containing vaterite implanted subcutaneously in BALB\c mice for its biocompatibility, integration pattern and immune response. Initially, a histopathological analysis was conducted. Furthermore, previously described histomorphometrical measurement methods were applied to analyze the immune response by quantification of M1 and M2 macrophages within the implantation beds of both IBS [30]. In addition, the in vivo transformation of vaterite into cHA was evaluated using electron-dispersive X-ray (EDX) mapping with scanning electron microscopy (SEM).

## 2. Results

### 2.1. Histopathological Results

**Figure 1:**
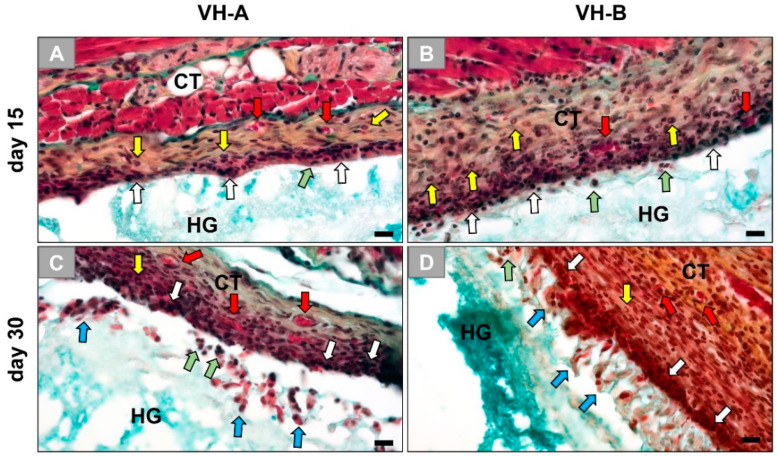
Exemplary histological images of the subcutaneously implanted hydrogels (VH-A and VH-B) at day 15 and 30 post implantation. (**A**) VH-A hydrogel and (**B**) VH-B hydrogel at day 15. (**C**) VH-A hydrogel and (**D**) VH-B hydrogel at day 30. CT = connective tissue, HG = hydrogel, white arrows = macrophages, green arrows = neutrophils, yellow arrows = eosinophils, red arrows = blood vessels and blue arrows = infiltration cells of the reactive tissue (Movat Pentachrome stainings, magnification = 400×, scalebars = 20 µm).

The histopathological analysis revealed that both compositions of the materials implanted induced minor inflammatory tissue reactions within the subcutaneous connective tissue at day 15 and 30 post implantation (Figure 1). Starting with day 15 post implantation, a cell-rich layer was found attached to the surface in both study groups that was mainly composed of macrophages in addition to lower numbers of eosinophilic and neutrophilic granulocytes (Figure 1A,B). Within the connective tissue adjacent to the biomaterials, blood vessels were also visible in both groups (Figure 1A,B).

At day 30 post implantation, the hydrogels still showed a minor tissue reaction that was mainly composed of macrophages and granulocytes (i.e., neutrophils and eosinophils) (Figure 1C,D). However, in both material groups, the reactive tissue appeared to have begun infiltrating the surfaces of the hydrogels with an apparent macrophage-mediated resorption of the hydrogels (Figure 1C,D).

**Figure 2:**
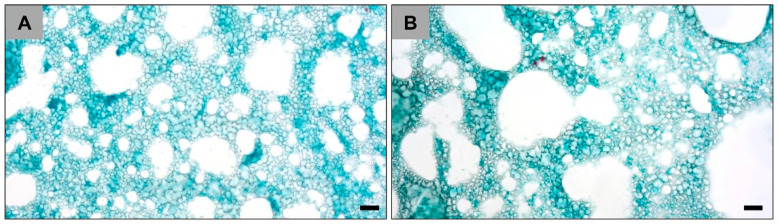
Exemplary microscopic images of the vaterite-loaded hydrogels in vivo within the implant bed centers at day 15 post implantation. (**A**) VH-A hydrogel. (**B**) VH-B hydrogel. (Movat Pentachrome stainings, magnification = 400×, and scalebars = 20 µm).

The histological analysis showed the microstructure of both hydrogels in vivo at each study time point (Figure 2). Both hydrogels appear to exhibit porosities but at different levels. The VH-A hydrogel seems to have higher porosity but smaller pore size and, in contrast, the VH-B hydrogel exhibited lower porosity but larger pore size (Figure 2). Furthermore, the VH-B group appears to have higher content and better distribution of vaterite particles.

**Figure 3:**
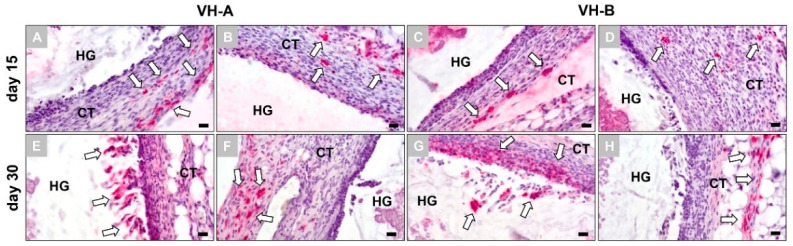
Immunohistochemical detection of pro- and anti-inflammatory macrophage subtypes at 15 and 30 days post implantation. (**A**) VH-A hydrogel at day 15, M1 immunostaining. (**B**) VH-A hydrogel at day 15, M2 immunostaining. (**C**) VH-B hydrogel at day 15, M1 immunostaining. (**D**) VH-B hydrogel at day 15, M2 immunohistochemical staining. (**E**) VH-A hydrogel at day 30, M1 immunostaining. (**F**) VH-A hydrogel at day 30, M2 immunostaining. (**G**) VH-B hydrogel at day 30, M1 immunostaining. (**H**) VH-B hydrogel at day 30, M2 immunostaining. White arrows: immunohistochemically stained macrophages, HG: hydrogel, CT: connective tissue (CD11c and CD163 immunostainings, magnification = 400×, scalebars = 20 µm).

The immunohistochemical detection, identification and location of the macrophage subtypes showed that both subforms were observable at a distinct distance from the material surfaces at 15 days post implantation (Figure 3A–D). However, the histological analysis did not show numerical differences between the different subforms in both groups and also no differences between the groups (Figure 3A–D).

At 30 days post implantation the analysis showed that markers for the pro-inflammatory macrophages were found at the material surfaces and especially by the cells invading both hydrogels (Figure 3E,G). In contrast, anti-inflammatory macrophages were still observed within the surrounding reactive tissue but at a distinct distance from the material surfaces (Figure 3F,H). Furthermore, no quantitative differences in the two immune cell subtypes were observed within and between the two study groups (Figure 3E–H).

### 2.2. Histomorphometrical Results

**Figure 4:**
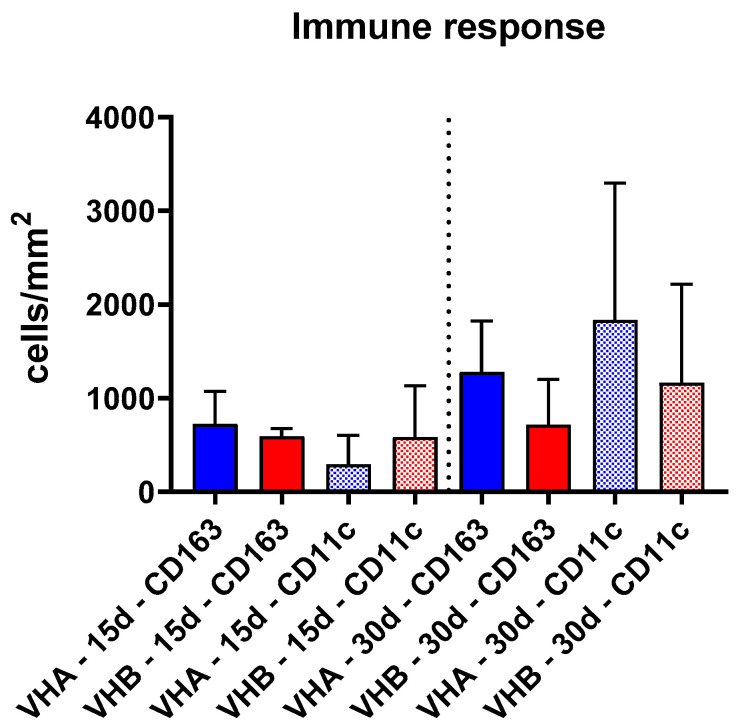
Results of the histomorphometrical analysis of the M1 and M2 macrophages within the implantation sites of both hydrogels (colored bars = anti-inflammatory cells, riffled bars = pro-inflammatory cells).

The quantitative analysis of pro- and anti-inflammatory macrophages within the implantation beds of the biomaterials showed that the number of CD163-positive M2 cells at day 15 post implantation was 726.2 ± 345.2 cells/mm^2^ and 593.4 ± 83.83 cells/mm^2^ for the VH-A and VH-B hydrogels, respectively (Figure 4). CD11c-positive M1 cells were present at 292.3 ± 311.9 cells/mm^2^ and 584.8 ± 549.5 cells/mm^2^ for the VH-A and VH-B hydrogels, respectively, at day 15 post implantation (Figure 4).

At day 30 post implantation, the number of CD163-postive M2 cells were found to be 543.1 ± 271.5 cells/mm^2^ and 481.8 ± 278.2 cells/mm^2^ for the VH-A and VH-B hydrogels, respectively (Figure 4). The numbers of CD11c-postive M1 cells appeared increased at 30 days post implantation. In the VH-A samples, the number of M1 cells were found to be 1458 ± 729 cells/mm^2^ and in the VH-B samples the number was 1051 ± 525.5 cells/mm^2^ (Figure 4). At both timepoints, the differences between the number of macrophages did not show any significant differences (Figure 4).

### 2.3. SEM/EDX Results

**Figure 5:**
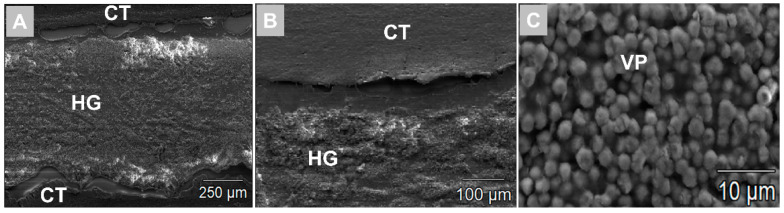
Exemplary SEM images from the VH-B group at day 30 post implantation. (**A**) Vaterite-loaded hydrogel (HG) within the subcutaneous connective tissue (CT) (magnification = 200×, scalebar = 250 µm). (**B**) Image from the hydrogel (HG)–tissue interface (magnification = 500×, scalebar = 100 µm). (**C**) Vaterite particles (VP) within the hydrogel region (magnification = 5000×, scalebar = 10 µm).

The analysis using scanning electron microscopy (SEM) revealed that both hydrogels maintained their bulk structure at both post implantation time points within the subcutaneous connective tissue (Figure 5A,B). Moreover, it could be shown that the vaterite particles were detectable within the implant beds (Figure 5C). The concentration range of the tested hydrogels was confined to 8% and 10%. No form-stable gels were obtained for concentrations <8%, and the porosity was no longer sufficient for concentrations >10%.

**Figure 6:**
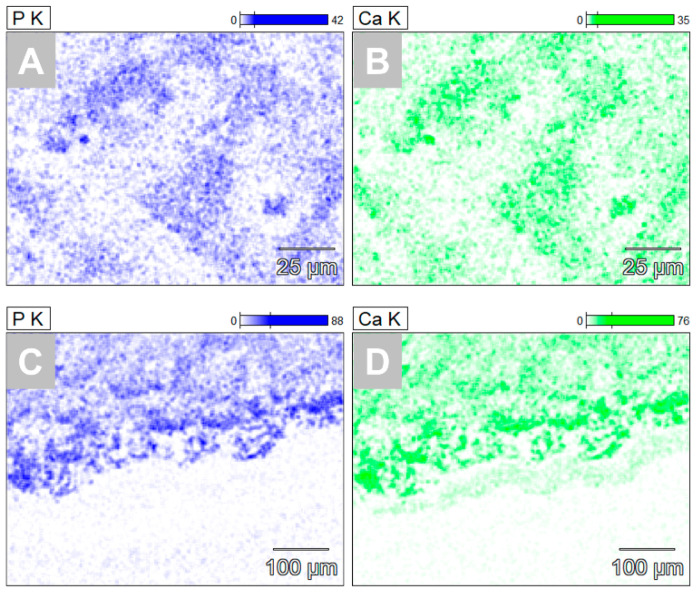
Exemplary SEM-EDX visualization of calcium (Ca) and phosphate (P) within the VH-A implantation beds at day 30 post implantation. (**A**) and (**C**) Phosphate presence, (**B**) and (**D**) calcium presence within the region of the implanted hydrogel up to the borders of the implantation beds (magnifications = 500×, scalebars = 100 µm).

The analysis using energy-dispersive X-ray spectroscopy (EDX) allowed a visualization of the elemental composition of the hydrogels and the surrounding tissue (Figure 6). Only calcium but no phosphate was detected within the implantation beds of both hydrogels at day 15 post implantation. At day 30 post implantation both calcium and phosphate were detected especially within the implantation beds of both hydrogels. No visible differences were observed between the two different materials (Figure 6). Both elements were found in the same overlapping locations within the implantation beds (Figure 6).

**Figure 7:**
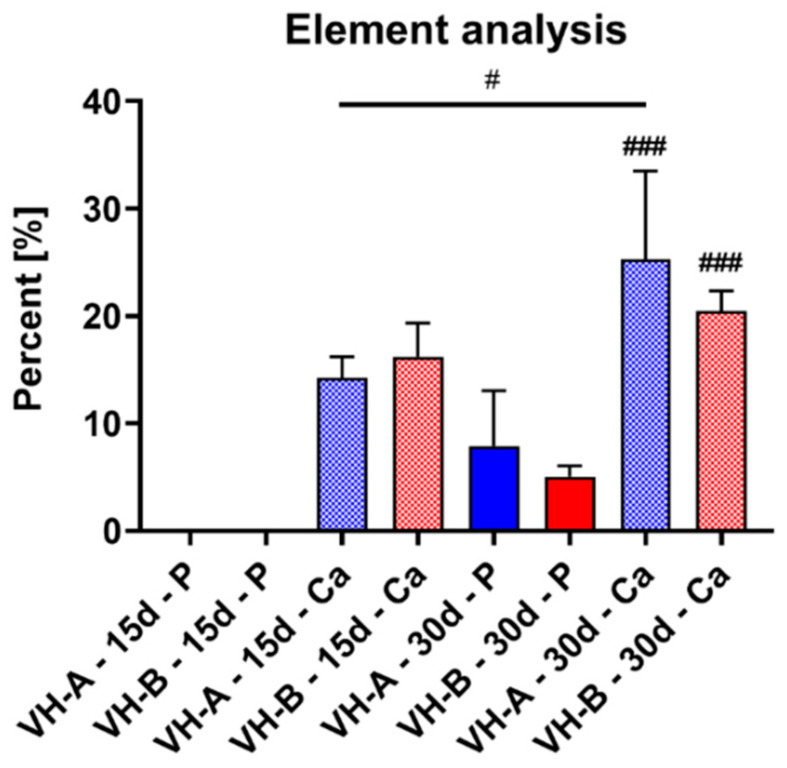
Analysis results of the percentages of calcium and phosphate ions within the implantation beds of both hydrogels hydrogels (colored bars = phosphate ions, riffled bars = calcium ions) (# = intra-individual significances, ^#^ *p* ≤ 0.05, ^###^ *p* ≤ 0.001).

The quantitative element analysis also showed that phosphate was not detected in the implantation beds of both study groups at day 15. However, at 30 days post implantation both calcium and phosphate were found in the implantation beds (Figure 7). Thereby, no significant differences were found analyzing the amounts of both calcium and phosphate in either of the materials containing vaterite at both time points (Figure 7). However, the measurements revealed that the calcium percentage was significantly higher than phosphate (^###^ *p* ≤ 0.001) for both groups at day 30 post implantation (Figure 7). In addition, the percentage of calcium appeared to increase from day 15 to day 30 in both hydrogel groups but was only significant for the VH-A materials (^#^ *p* < 0.05) (Figure 7).

## 3. Discussion

The aim of the present study was to investigate different ratios of poly(ethylene glycol)-acetal-dimethacrylat (PEG-a-DMA) hydrogels with vaterite, i.e., 8% (*w*/*v*) (VH-A) or 10% (*w*/*v*) (VH-B), for its biocompatibility, integration pattern and immune response. In this context, the application of IBS has been extended in the last decades to include not only the field of traumatology or orthopedics but also in dentistry, as this material class allows for a minimally invasive insertion in addition to a precise filling of the defect cavity [31]. In this case, an optimal IBS should function as osteoconductive scaffolds while being integrated and degraded over time following the principle of “creeping substitution” [32]. As an example, preclinical in vivo studies and also clinical studies analyzing the tissue reactions and integration behavior of an IBS based on β-TCP granules and a hydrogel based on modified cellulose and sodium hyaluronate as well as an IBS composed of a water-based gel combined with nano-hydroxyapatite (HA) particles and biphasic calcium phosphate granules showed that this material class allows for an integration and degradation pattern moving from the periphery to the implant center according to the concept of Guided Bone Regeneration (GBR) [25,27,33]. Thus, the aim of the present study was to utilize a subcutaneous implantation model in combination with established and standardized analysis methods to compare the tissue reaction of a newly developed IBS to the results obtained with other materials in previous studies using equivalent methods [25,27,33].

Initially, the histopathological analysis revealed that both IBS compositions induced minor inflammatory tissue reactions within the subcutaneous connective tissue at days 15 and 30 post implantation. Interestingly, the materials were found en bloc, i.e., completely coherent and with signs of fragmentation, within their implantation beds at both time points. While a cell-rich layer was found attached to the surface in both study groups at day 15 post implantation, a slight reactive tissue appeared to have begun infiltrating the surfaces of the hydrogels with an apparent macrophage-mediated resorption of the hydrogels in both material groups at day 30 post implantation. This integration behavior is completely different from what was found in the previously described studies that showed that the reactive tissue infiltrated the interspaces of the tissue containing BSM granules [25,27,33]. In the case of both calcium phosphate-based IBS the cellular infiltration was observed in some cases at day 3 and in all at day 10 post implantation, and this continued in a stepwise time-dependent manner into the center of the implanted material. Macrophages, biomaterial-associated multinucleated giant cell (BMGCs) and complex tissue including high numbers of vessels were observed as parts of the tissue reactions. Although both materials were biphasic the phagocytes were shown to primarily penetrate the hydrogel phases, i.e., cellulose and hyaluronate as well as the water-base nano-HA gel, and to penetrate the granule interspaces.

In the present study, the mixtures of the PEG-a-DMA-based hydrogels with vaterite particles appeared to have formed an overall compound that only allowed a superficial cell penetration up to day 30 post implantation. This observation indicates a very slow integration of the two biomaterials—although the inner core of the two materials had a similar porous structure at these early time points. Thus, it can be assumed that a connective tissue and subsequent bony integration would only take place at a much later point in time. This assumption is further supported by the fact that only mononuclear cells or macrophages were found on the material surfaces. From previous studies, it is known that the phagocytosis capacity of these phagocytes seems to be much smaller even in comparison to their fused end stage, the BMGC [34]. The conclusion from this observation is that the lower a material’s ability to induce BMGCs is, the lower the rate of material degradation is also observed in most cases. This observation is furthermore supported by the phagocytosis capacity of BMGCs. Phagocytosis is often higher in BMGCs than in their mononuclear precursors that have engulfed fragments with a diameter of ~500 µm as was shown previously for a synthetic BSM [12,35]. Although it was not possible to measure the phagocytized material, it has been demonstrated that the fusion process of monocytes or macrophages is induced by the size of a foreign bodies. This leads to a condition where the membrane capacity required for the uptake is exceeded in addition to other underlying molecular biological factors [15].

However, the question in this case—and also in case of other different biomaterials—is why oversized materials such as the combination of the hydrogels and the vaterite particles did not induce the fusion to BMGCs. As has been shown in other studies, the sum of the physicochemical properties of a biomaterial on macrophages is not well known and there is a complex interplay between biomaterial properties and those that result from interactions with the local environment [15,17]. However, the fusion factors even in the context of the foreign body response to biomaterials have not been well characterized. In brief, it has been shown that calcium ions binding to Soluble NSF Attachment protein REceptors (SNAREs) induce cell–cell fusion [36]. Furthermore, monocyte/macrophage fusion is also assumed to occur via induction of membrane lipid rafts and a related exposure of adhesion molecules, via actin polymerization with the involvement of t-SNAREs [36,37]. In addition, the involvement of pseudopodia/filopodia that have shown to be involved in processes such as chemotactic sensing of the environment, controlling the direction of cell migration and substrate adhesion, are fusion factors of BMGCs [38,39]. Finally, soluble mediators such as IL-4 or IL-13 are known factors of the BMGC formation [40]. None of these factors appeared to be present in the case of the materials examined in the present study. One explanation for this observation might be that smaller subunits of the hydrogel/vaterite combination may have been phagocytosed by macrophages that were found in high numbers at the material surfaces. Another explanation is the cellular migration into the materials, which supports this supposition. However, further studies are necessary to elucidate the degradation mechanisms of hydrogel/vaterite biomaterials with a special focus on the involvement of both macrophages and BMGCs.

A further observation in the present study was the localized presence of macrophage subtypes. The pro-inflammatory subtypes were found accumulated at the material surfaces and the anti-inflammatory macrophages were predominantly found within the surrounding connective tissue with a clearly visible distance to the implants. This observation on the one hand supports a previously reported hypothesis that described the involvement and necessity of M1 macrophages for the biodegradation of biomaterials similar to those described in the present study [41,42]. In this context, the M1 macrophages that appeared to be involved in the degradation of the materials in the present study—in clear differentiation to the M2 macrophages—may express lytic enzymes such as the members of the group of reactive oxygen species (ROS) and other relevant mediators [19]. Thus, the results of the present study support the thesis that the biodegradation of the actual BSM is mediated by “(pro-)inflammatory cells” and is not a physiological process, which indicates that inflammation is needed in order for degradation to proceed. However, the term “inflammation” should not be misunderstood or interpreted negatively in this context, as the histomorphometrical results overall show a balancing relationship between pro- and anti-inflammatory macrophage subtypes. Altogether, this indicates a local inflammatory homeostasis of the tissue and a biocompatibility of the two biomaterials investigated without any differences observed between both study groups.

The in vivo transformation of vaterite into cHA was evaluated using electron-dispersive X-ray (EDX) mapping with scanning electron microscopy (SEM) as previously described [43]. This analysis revealed that only calcium, but no phosphate, was detected within the implantation beds of both hydrogels at day 15 post implantation. In contrast, both calcium and phosphate were detected primarily within the implantation beds of both hydrogels at day 30 post implantation. In addition, both elements were found in the same overlapping locations within the implantation beds. No visible differences were observed between the two different study groups. The quantitative element analysis confirmed the qualitative observations as only calcium was detected at day 15 post implantation, while both calcium and phosphate were measured in comparable amounts in the implantation beds of both IBS at day 30 post implantation. The amount of calcium was significantly higher, i.e., three times higher than that of phosphate, which corresponds to the stoichiometric distribution ratio of calcium phosphates [44]. Thus, tricalcium phosphate (Ca_3_(PO_4_)_2_) has an exact CaO/P_2_O_5_ ratio of 3:1 and hydroxyapatite (Ca_5_(PO_4_)_3_OH) a slightly higher calcium content [44]. Moreover, only a significant increase in the HA content was found in the V-HA group.

These data indicate that the vaterite in the IBS in this study most likely underwent a transformation into calcium phosphate. This presumably occurs via a stepwise transformation as only calcium was detectable in measurable levels within the outer regions of implantation beds. This is the area in which the materials mainly interact with the surrounding body environment, i.e., with the interstitial body fluid which acts as a “polar organic solvent”. Thus, it appears that the vaterite, a metastable form of calcium carbonate with a high solubility, was converted initially to calcite (as the most stable form of calcium carbonate) as this process takes place once vaterite is exposed to water [45]. Thereby, the vaterite dissolves and subsequently precipitates as calcite assisted by an Ostwald ripening process at 37 °C [46,47]. This could be the reason for the initial detection of calcium at day 15 post implantation. Phosphate is then attracted by the positive surface charge and presumably interacts with the calcium [23]. This was measured at day 30 post implantation, indicating a natural bone-like in vivo mineralization. However, longer implantations are needed in order to determine whether this progresses with time. Although this observation confirms the in vivo conversion to bone-like material of the vaterite-containing IBS, it shows that the observation period of the present study was not sufficient. Further studies are needed to determine the time span needed for a complete transformation of the vaterite precursor material. Furthermore, the question arises if the observed slow conversion behavior of the IBS is suitable for bone tissue regeneration. However, in vitro results studies have shown that vaterite in hydrogels is able to serve as a substrate for adhesion and as a mineral substrate for natural bone formation by osteoblasts [48].

The results of the present study demonstrated that both of the vaterite-containing hydrogels differing only slightly in the PEG-acetal-DMA content are fully biocompatible. The resulting scaffolds were degraded in vivo by mononuclear cells of the macrophage lineage via a pro-inflammatory process and resulted in a balanced occurrence of M1 and M2 macrophages which additionally underlines the biocompatibility of the material. Furthermore, a stepwise transformation of the vaterite into calcium phosphates was measured and indicates that vaterite particles may be a highly favorable compound for use in bone regeneration applications. Finally, the novelty of this study is that the present results could show the integration behavior of newly developed IBS composed of vaterite nanoparticles with special focus on the immune responses and the observed conversion of vaterite into calcium phosphate in vivo.

Altogether, it can be concluded based on the present preclinical study results, that both tested biomaterials are suitable for application in clinical (dental) indications such as socket preservation or sinus augmentation, but also in the field of orthopedics such as spinal fusion. This assumption is based on the fact that vaterite provides good structural properties including a sufficient porosity in vivo, which is known to allow for the long-term ingrowth of stable vascularization and finally of bone tissue.

## 4. Materials and Methods

### 4.1. Biomaterial Preparation

#### 4.1.1. Vaterite Nanoparticles

Preparation was carried out according to a previous publication [21]. Briefly, 5 mmol of calcium chloride tetrahydrate was dissolved in 50 mL ethylene glycol by sonication at 40 C (Emmi 40 HC by EMAG-Technologies, at max. power, frequency, and ultrasonic power: 250 W, 45 kHz and 100%, respectively). An amount of 10 mmol of sodium bicarbonate was dissolved in 50 mL ethylene glycol via mechanical stirring. The solutions were mixed and sonicated for 25 min at 40 °C. Afterwards, 50 mL of sterile water was added, and the mixture was sonicated for an additional 5 min. The mixture was centrifuged at 9000 rpm for 30 min and the CaCO_3_ precipitated was decanted from the turbid, washed with water/ethanol and vacuum dried.

#### 4.1.2. Vaterite-Loaded PEG Hydrogel

Preparation of PEG-acetal-DMA precursor was carried out as previously described [21]. Briefly, 1 mmol of PEG8000, 5 mmol 2-(vinyloxy)ethyl methacrylate and 0.1 mmol *p*-toluene sulfonic acid were dissolved in 5 mL dichloromethane (20 mg of hydroquinone was added as a radical inhibitor and 0.2 mmol triethylamine as a quencher were added). The reaction was followed by extraction and vacuum drying. To synthesize the hydrogel, 8% (*w*/*v*) or 10% (*w*/*v*) PEG-acetal-DMA was dissolved in DPBS. In addition, 1% vaterite was added to the solution in each case and homogenized by ultrasound. For cross-linking, 0.2% (*w*/*v*) of the photoinitiator 2-hydroxy-4′-(2-hydroxyethoxy)-2-methylpropiophenone was added as a 10% (*w*/*v*) solution in ethanol (70%). Polymerization was finally initiated at 365 nm UV irradiation for 15 min in a 24-well plate.

### 4.2. Experimental Animals and Surgical Procedure

Following the authorization from the Local Ethical Committee (number of approval: 323-07-00278/2017-05/6; date of approval: 13 July 2017), in vivo implantation of the hydrogels was carried out. The experimental preclinical procedure was carried out at the Faculty of Medicine in the University of Niš (Serbia). Animal acclimation was carried out using standard conditions (i.e., water ad libitum, artificial light, and regular rat pellets). The animals were treated similarly with standard pre- and post-operative care.

A total of 20 female BALB/c mice were obtained for the studies from the Military Medical Academy (Belgrade, Serbia) and these were randomly assigned in two groups (VH-A and VH-B). Each group contained 10 experimental animals, 5 for each timepoint (15 and 30 days). The implantation was carried out according to a protocol described by Barbeck et al. [18,33,49,50,51,52]. Briefly, the animals were anesthetized via an intraperitoneal injection (10 mL ketamine (50 mg/mL) with 1.6 mL Xylazine (2%)). On the shaved area of the skin, an incision down to the subcutaneous tissue within the rostral subscapular region was made. Subsequently, a subcutaneous pocket was bluntly built by a scissor and the biomaterials were injected into the pocket. Afterwards, the wounds were sutured.

At the respective timepoints (15 and 30 days), the mice were euthanized using an overdose of the above-mentioned anesthetics and the implantation area with its surrounding tissue were explanted. Subsequently, the explanted tissue was fixed using a 4% formalin solution for 24 h and then placed into PBS prior to additional processing.

### 4.3. Histological Workup

The preparation for histological staining began with cutting the explants into two dimensionally identical segments and was then followed by stepwise dehydration using increasing alcohol concentrations. The segments were embedded in paraffin after exposure to xylene. Sections with 3–5 µm thickness were then cut using a rotation microtome (SLEE, Mainz, Germany) from the segments. Two sections of every explant were used for histochemical staining (i.e., hematoxylin and eosin staining (HE) plus Movat Pentachrome staining).

Two additional sections of each tissue explant were used for immunohistochemical detection of macrophages. CD11c-positive M1 macrophages and CD163-postive M2 macrophages were stained using antibodies against the pro- and anti-inflammatory molecules based on previously published methods [53]. Initially, the sections were treated with citrate buffer and proteinase K in a heated water bath for 20 min at 96 °C. Subsequently, the slides were subjected to H_2_O_2_ and avidin and biotin blocking solutions (Avidin/Biotin Blocking Kit, Vector Laboratories, US). After this, the sections were incubated with the respective primary antibody (CD163 or CD11c) for 90 min which was followed by incubation with the secondary antibody (goat anti-rabbit IgG-B, sc-2040, 1:400, Santa Cruz Biotechnology, Dallas, TX, USA). After the incubation period, the avidin-biotin-peroxidase complex (ThermoFisher Scientific, Waltham, MA, USA) was applied for 60 min and were counterstained with hemalum.

### 4.4. Histological Analysis

To examine and analyze the histological staining of the tissue–biomaterial interaction within the implantation bed, an Axio Imager M2 (Zeiss, Oberkochen, Germany) was used based on a protocol according to the DIN ISO 10993-6 as previously described [35,54,55,56,57]. These analyses focused on the qualitative evaluation of specific parameters within the framework of early and late tissue response to medical devices. The parameters are fibrosis, hemorrhage, necrosis, vascularization, and tissue granulation. Thus, the evaluation included the detection of several cell types: granulocytes, lymphocytes, plasma cells, macrophages and biomaterial-associated multinucleated giant cells (BMGCs). Finally, microphotographs were taken with an Axiocam 506 color connected to a computer system running the ZEN Core (Zeiss, Oberkochen, Germany) connected to the microscope.

### 4.5. Histomorphometrical Analysis

The histomorphometrical analyses included quantitative measurements of the presence of anti-inflammatory and pro-inflammatory cells within the implant beds of the hydrogels as previously described [58]. Briefly, the immunohistochemical-stained tissue sections, as described above, were digitized by a specialized scanning microscope (PreciPoint M8, PreciPoint GmbH, Freising, Germany). After this, the Image J software was used to measure the stained cells within the total scans. At first the total area and the hydrogel area were manually marked, and their areas were determined. After this, the number of macrophages was also determined using a specially programmed plugin that automatically identified and marked the area of the red stained cells [30]. As a final step, the cell numbers were correlated to the respective total area to calculate the numbers of cells per mm^2^ (macrophages/mm^2^).

### 4.6. SEM/EDX Analysis

The in vivo SEM/EDX analysis for illustration and measurements was conducted as previously described by Jung et al. [43]. In brief, the element distribution analysis was conducted via a LEO Gemini 1530 with a field-emission gun (Carl Zeiss AG, Jena, Germany). Therefore, the trans-sections were initially precoated using carbon and immediately transferred to scanning electron microscopy (SEM) and energy-dispersive X-ray spectroscopy (EDX). The EDX maps (256 × 196 pixels) were recorded using a Thermo Noran X-ray detector in combination with the ThermoFisher Scientific software Noran System Six. Therefore, voltage was set to 5 kV for imaging and 10 kV for EDX mapping.

### 4.7. Statistical Analysis

Quantitative data are shown as mean ± standard deviation after an analysis of variance (ANOVA), which enabled a comparison of the data from the study groups via the GraphPad Prism 8.0 software (GraphPad Software Inc., La Jolla, CA, USA). Statistical differences were designated as significant if *p*-values were less than 0.05 (^#^ *p* ≤ 0.05), and highly significant if *p*-values were less than 0.01 (^##^ *p* ≤ 0.01), less than 0.001 (^###^ *p* ≤ 0.001) and less than 0.0001 (^####^ *p* ≤ 0.0001).

## Data Availability

All data are included in the manuscript.
